# Resilience in Environmental Risk and Impact Assessment: Concepts and Measurement

**DOI:** 10.1007/s00128-018-2467-5

**Published:** 2018-10-24

**Authors:** David G. Angeler, Craig R. Allen, Ahjond Garmestani, Kevin L. Pope, Dirac Twidwell, Mirco Bundschuh

**Affiliations:** 1 Department of Aquatic Sciences and Assessment, Swedish University of Agricultural Sciences, Uppsala, Sweden; 2 School of Natural Resources, University of Nebraska – Lincoln, Lincoln, NE USA; 3 U.S. Geological Survey, Nebraska Cooperative Fish and Wildlife Unit, School of Natural Resources, University of Nebraska – Lincoln, Lincoln, NE 68583 USA; 4 National Risk Management Research Laboratory, U.S. Environmental Protection Agency, Cincinnati, OH 45268 USA; 5 Department of Agronomy and Horticulture, University of Nebraska – Lincoln, Lincoln, NE 68503-0984 USA; 6 Institute for Environmental Sciences, University of Koblenz – Landau, Landau Campus, 76829 Landau, Germany

## Abstract

Different resilience concepts have different assumptions about system dynamics, which has implications for resilience-based environmental risk and impact assessment. Engineering resilience (recovery) dominates in the risk assessment literature but this definition does not account for the possibility of ecosystems to exist in multiple regimes. In this paper we discuss resilience concepts and quantification methods. Specifically, we discuss when a system fails to show engineering resilience after disturbances, indicating a shift to a potentially undesired regime. We show quantification methods that can assess the stability of this new regime to inform managers about possibilities to transform the system to a more desired regime. We point out the usefulness of an adaptive inference, modelling and management approach that is based on reiterative testing of hypothesis. This process facilitates learning about, and reduces uncertainty arising from risk and impact.

There have been many recent calls for assessing ecosystem resilience to improve management and conservation and to assess risks posed to ecosystems due to human activity (e.g., Curtin and Parker 2014; Standish et al. 2014; Bundschuh et al. 2017). The many forms of environmental pressures (e.g., agriculture, land-use and climate change) that rapidly change current ecological baselines highlight the pressing nature of this problem. Ecologists and managers are aware that the capacity of ecosystems to adapt to environmental change may be exhausted in the future. This may lead to a profound reorganization of ecosystems at local, regional and planetary scales (Hughes et al. 2013). However, predicting and assessing ecosystem change in relation to risks and how this affects ecosystem service provisioning is fraught with uncertainty.

Because of the pervasive problems that threaten the world’s ecosystems, scientists and managers are increasingly interested in using resilience concepts in environmental risk and impact assessment. The term resilience has become commonplace as a boundary concept in social, health, technological and ecological sciences (Brand and Jax 2007; Baggio et al. 2015). In each science, multiple definitions of resilience have been proposed and debated (Myers-Smith et al. 2012). However, with the diversification of the term, its clarity has often been lost (Brand and Jax 2007). In ecology, the term resilience has been used interchangeably in at least two different contexts, each based on distinct assumptions of *alternative system regimes* (for definitions of terms in italics see Table 1) (Angeler and Allen 2016). That is, on one hand resilience can be understood as recovery from disturbances, the prevalent concept used in the ecological sciences. On the other hand, resilience describes substantial reorganizations when a disturbance threshold is passed and the system stabilizes in a new regime from which it will not recover to a previous regime. Accounting for such assumptions has significant implications for resilience-based risk assessment (Bundschuh et al. 2017). In this paper, we discuss these two resilience concepts and measurement approaches.

**Table 1:** Definitions of terms used in this paper

• Adaptive capacity: Latent property of an ecological system (or other complex system) to respond to disturbances in a manner that maintains the system within its current basin of attraction by altering the depth and/or width of that basin (e.g., the shape of cups in the ball-in-cup heuristic; Fig. 1)
• Ecological resilience: A measure of the amount of change needed to change an ecosystem from one set of processes and structures to a different set of processes and structures (e.g., change of grasslands to agriculture to wastelands; the American dustbowl in the 1930s)
• Engineering resilience: Return time to equilibrium after disturbance (e.g., a phytoplankton community recovering from a herbicide pulse)
• Alternative system regimes: A potential alternate configuration in terms of the structural and functional composition, processes, and feedbacks of a system (i.e. the two cups in Fig. 1 exemplify alternative regimes, which in nature can be a turbid vs a clear-water shallow lake)
• Basin of attraction (stability domain): A region of the state space where the system tends to remain and has a definable configuration in terms of the abundance, composition, and processes of a system (i.e. the complex interaction of abiotic and biotic factors that shape a system regime)
• Cross-scale resilience: The degree to which a system has high functional diversity and high functional redundancy within and across the scales of an ecosystem. Cross-scale resilience accounts for the hierarchical organization of ecosystems
• Stability: A system characteristic whereby system properties remain unchanged within a basin of attraction following disturbance. Stability has therefore a single equilibrium focus. The wider and deeper the basin of attraction, the higher its stability
• Persistence: Duration of species existence before it becomes extinct (either locally or globally)
• Resistance: The external force or pressure needed to displace a system by a certain amount
• Variability: Inverse of ecological stability; fluctuation in ecosystem parameters over time
• Functional diversity: Diversity of reproductive phenology, seed bank potential, colonization and dispersal abilities, and other traits
• Functional redundancy: Existence of more than one species or process delivering the same ecological function. This contributes to adaptive capacity in ecosystems by providing buffering for loss of function due to disturbance-induced mortality
• Response diversity: Variability among individuals or species in the range of response patterns to disturbances, which depends on the composition and expression of multiple functional traits of organisms (e.g., high-dispersal, fast growth; high-dispersal, low growth; low-dispersal, fast growth; low-dispersal, low growth)
• Regime shift: Persistent change in structure, function, and feedbacks of an ecosystem
• Thresholds: Point upon which the capacity of a system to absorb disturbances is exhausted which leads to the reorganization of the system in a new alternative regime; that is, when the system undergoes a regime shift

**Fig. 1:**
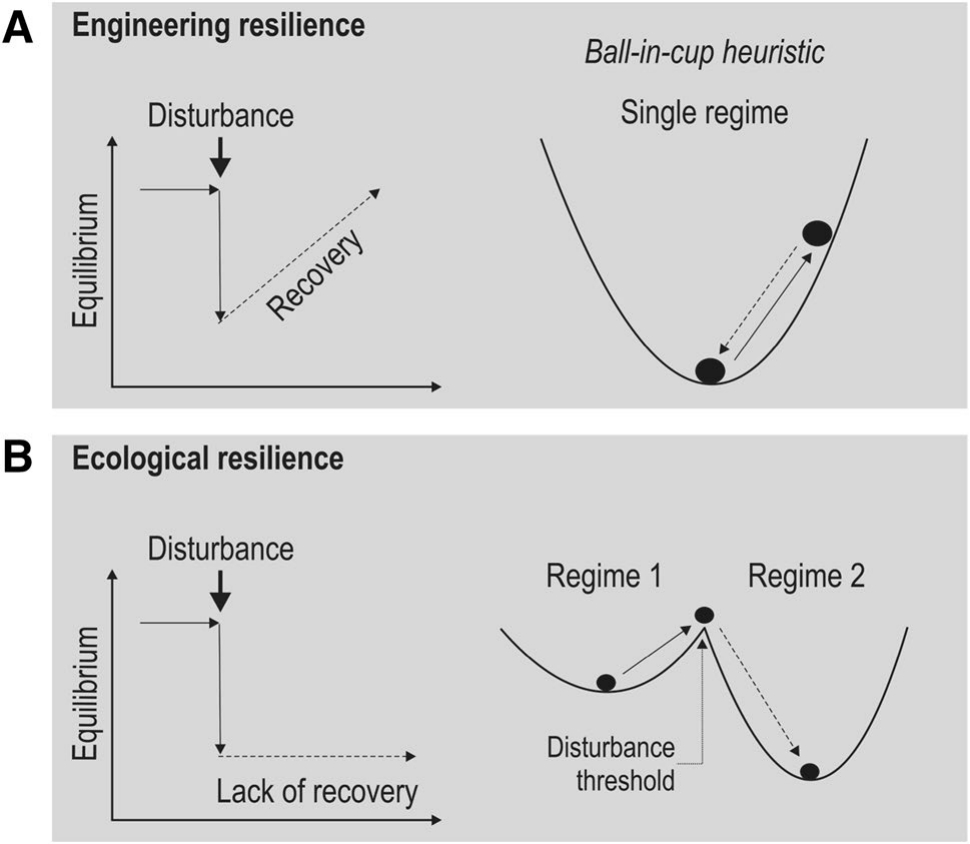
Schematic distinguishing between **a** recovery (engineering resilience) and **b** ecological resilience. Panels on the left show ecosystem trajectories before, during, and after disturbances. Panels on the right express these dynamics with ball-in-cup heuristics commonly used in ecology. In the case of recovery/engineering resilience, the ball rolls back to its equilibrium position after a disturbance. In the case of ecological resilience, the ball rolls over the cup’s brink and falls into a new cup. This cup represents an alternative stable system regime from which recovery to the previous regime is impossible. This is symbolized with the ball not rolling back to the previous cup

## Concepts

There are currently two broad groups of scholars with different foci on resilience in the ecological sciences. Traditional ecological *stability* research presumes that ecological patterns and processes operate in a single equilibrium regime (*basin of attraction*). Many of the resilience concepts used in this context focus on *resistance, persistence, variability* and resilience (Donohue et al. 2013). Resilience in ecological stability research is often specified as *engineering resilience* to more clearly separate it from *ecological resilience* (Gunderson 2000). Engineering resilience is equal to the commonly used terms resiliency, recovery and bounce back (Angeler and Allen 2016).

Ecological stability measures are useful for characterizing responses after disturbances; that is how much ecosystems deviate from, fluctuate and recover after perturbations. However, these measures neither capture the complex adaptive systems behavior of ecosystems, which emerges from the complex interplay of abiotic and biotic factors, nor the existence of alternative regimes (e.g., lakes in a clear-water versus a turbid regime) (Allen et al. 2014). This complex adaptive systems behavior is accounted for in the definition that is now commonly known as ecological resilience (Gunderson 2000). This concept increasingly attracts scholars in natural sciences, including risk assessment (e.g., Bundschuh et al. 2017), and design for infrastructure and construction (Jennings et al. 2013). Ecological resilience is the magnitude of disturbance that can be absorbed (*adaptive capacity*) before the system passes a *threshold*, which leads to a substantial reorganization of its structure and functions and stabilization in an alternative regime (Gunderson and Holling 2002).

Despite differences between concepts, engineering resilience and ecological resilience are not mutually exclusive. Engineering resilience is subsumed within the broader systemic organization of ecosystems from which ecological resilience emanates. That is, engineering resilience can be observed within distinct alternative states as part of the broader dynamics of ecosystems. For example, fish communities may recover after a pesticide pulse, and this bounce back may occur in the turbid and clear-water alternative regimes of a lake. In both regimes, recovery results from the systemic properties that stabilize each regime. However, engineering resilience fails to describe shifts between regimes: once a lake has shifted to and locked in a turbid regime it will not recover to a clear-water regime due to self-reinforcing feedbacks.

Discerning resilience concepts has potential to contextualize risk and impact assessment. In the next sections we explore the relationships between engineering resilience and ecological resilience. Specifically, we discuss how both concepts can be quantified and inform risk and impact assessment.

## Measurement

The measurement of engineering resilience is relatively straightforward, using time to recover from a disturbance as the unit. Engineering resilience has important implication in a risk and impact assessment context, because recovery indicates a potential low long-term risk of stressors to fundamentally disrupt ecosystem structure and function. In turn, this could indicate that a system may continue to provide ecosystem services if this system is in a desired regime. However, research suggests that engineering resilience can slow down when disturbances push an ecosystem to a critical threshold, which in turn serves as a warning signal for profound system change (Scheffer et al. 2009). Once a threshold has been past, ecosystems fail to show recovery after a disturbance. In such a case, rather than rebounding, the system moves into an alternative regime (Fig. 1)—it has undergone a *regime shift*—which poses new challenges for risk and impact assessment. These challenges are related frequently to the high uncertainty regarding ecosystem service provisioning. It is therefore important for risk and impact assessment to understand ecosystem stability to identify management interventions to transform the system back into a more desired regime.

**Fig. 2:**
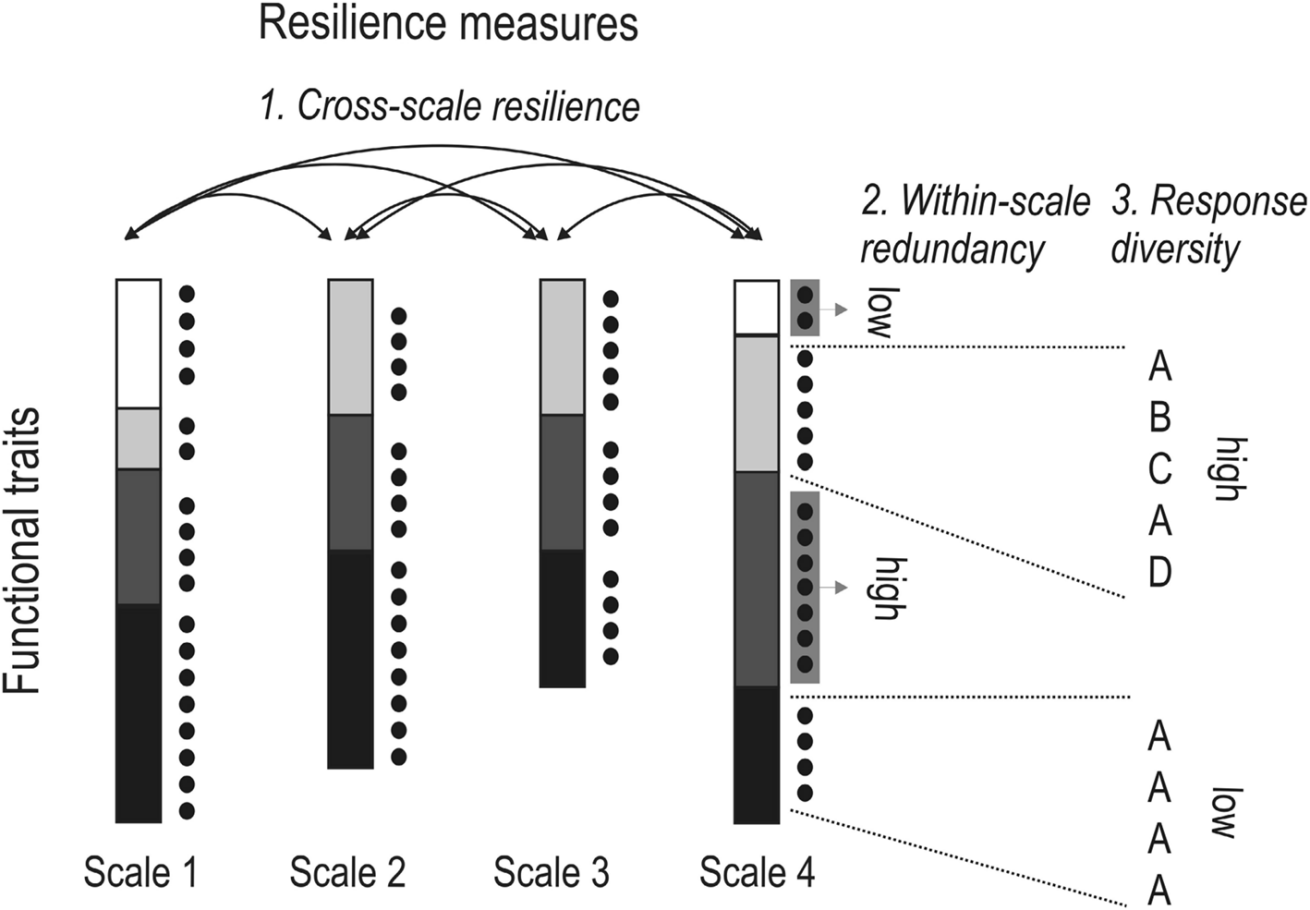
Cross scale resilience model modified from Angeler et al. (2016). For description see text

Understanding stability relationships, which influence the ecological resilience in ecosystem, requires taking into account system complexity. Recently, ecological resilience proxies that account for this complexity have been suggested for management (Angeler et al. 2016). These proxies are based on the *cross-scale resilience* model (Peterson et al. 1998) (Fig. 2), which can be used in various ways to support environmental impact and risk assessment of chemicals and other stressors (Bundschuh et al. 2017).

## Cross-Scale Resilience

A major tenet of the cross-scale resilience model is that it accounts for ecosystem complexity, which is manifested in the hierarchical organization of ecosystems and therefore explicitly incorporates scale (e.g., from local patches, to regions, to biomes, to the globe) (Nash et al. 2014). These scales can be objectively analyzed with different statistical approaches, for example using animal body size, an integrative variable that is allometric with many physiological and ecological attributes (Peters 1983). The cross-scale resilience model builds on quantifying biodiversity aspects at each of the identified scales in the ecosystems’ hierarchies. That is, it assesses *functional diversity* and *redundancy* of species in addition to their functional traits and abundances at each scale (Sundstrom et al. 2018). Examining the distribution of traits allows assessing how redundant they are within each scale (within-scale redundancy) or across the scales (cross-scale redundancy) present in the system (Fig. 2). This is relevant in the context of scrutinizing the impacts of disturbance. Disturbances in ecosystems are scale specific (a hail shower may have a significantly higher impact on seedlings compared to trees), so the higher the redundancy of functional traits within and across scales, the higher the ability of the ecosystem to cope with disturbances.

In addition to focusing on the redundancy of functional traits the cross-scale resilience model considers the variation in responses to environmental change by species within a functional group within scales (*response diversity;* Elmqvist et al. 2003). That is, response diversity considers the functional make up of a species accounting for multiple traits that modulate species responses through, for instance, distinct colonization, growth, competition, and dispersal abilities. Figure 2 provides an example of cross-scale resilience showing the distribution of functional traits (squares with different shades of grey) and the number of species (black dots) in each species group (redundancy) across scales. It shows low and high redundancy and response diversity. The latter is indicated by letters exemplifying variability in the composition of multiple functional traits of species (e.g., A = slow growth, low dispersal; B = fast growth, low dispersal; C = fast growth, high dispersal, D = slow growth, high dispersal) that confer different response potential of species to disturbances. Cross-scale resilience is indicated by arrows connecting individual scales. In this example, the white guild has the lowest cross-scale redundancy (Fig. 2).

In short, the cross-scale resilience model builds on the notion that ecological functions and processes, and ultimately ecosystem resilience, depend on the redundancy, distribution and diversity of functional traits of species within and across spatiotemporal scales. Measuring how these functions change within and across scales over time allows for an assessment of the relative resilience of a system (Allen et al. 2005). This can be useful for risk and impact assessment, particularly for evaluating the recovery potential after disturbances or the novel ecosystem characteristics that emerge once a disturbance threshold has been passed and the system organizes in a new, potentially degraded and undesired regime. In this case risk and impact assessment can focus on the identification of stability characteristics and inform management about possible interventions to transform the system to a more desired regime.

**Fig. 3:**
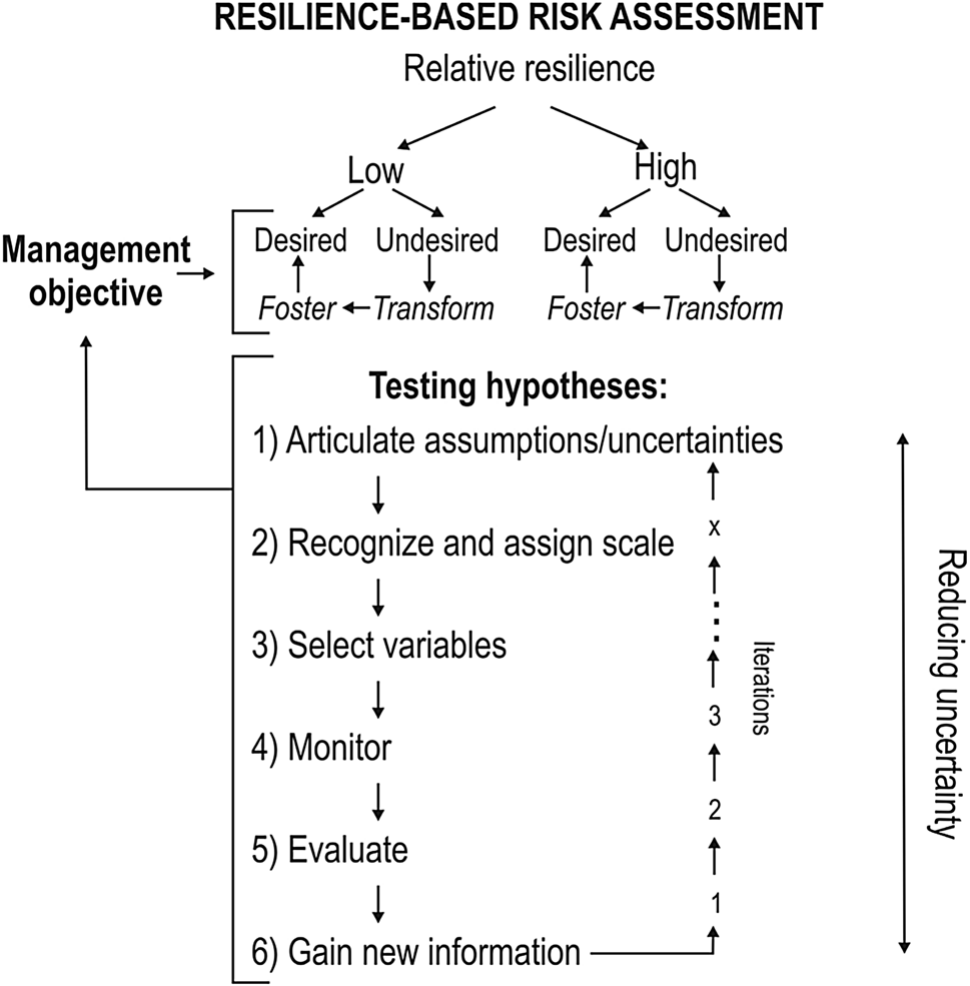
Schematic showing adaptive approaches for resilience-based environmental risk and impact assessment [Figure modified from Baho et al. (2017)]

Resilience-based impact assessment could, thus, begin with obtaining a snap shot of relative resilience by measuring the proxies of cross-scale resilience, within-scale redundancy and response diversity within terrestrial and aquatic animal communities (Nash et al. 2014). Measuring these proxies sequentially could provide insight into the persistence or change of the system over time and inform impact assessment (Fig. 3). Imagine a stream in an agricultural landscape in which eutrophication poses risks to aquatic ecosystem integrity. Resilience-based impact assessment could be geared towards assessing these risks and how the system should be managed for the maintenance of ecosystem services (clean water, fish for human consumption, recreation; e.g., Birgé et al. 2016; Pope et al. 2016). However, one major problem with risk assessment and management is the high uncertainty of outcomes; for instance, the efficiency of chemical-runoff and nutrient control. To reduce this uncertainty, managers could implement an adaptive management, inference and modeling framework (Baho et al. 2017). Specifically, managers can pose, based on available scientific information, hypotheses about risks stemming from several impact sources (e.g., sediments, nutrients, pesticides in a stream). Managers could then implement management actions and evaluate outcomes based on the verification or falsification of these hypotheses. Reiteratively testing, refining and recalibrating hypotheses about the impact of sources (i.e., the multiple stressors in the stream example above) over time ultimately has potential to incrementally decrease uncertainty in risk assessment and provides information about whether or not preventative or restorative measures and which types of interventions are most promising.

## Summary

There is abundant research from terrestrial (Allen et al. 2005; Fischer et al. 2007; Wardwell et al. 2008), aquatic (Baho et al. 2014; Angeler et al. 2015; Nash et al. 2016) and other complex systems (Sundstrom et al. 2014) that supports the cross-scale resilience model. Based on this evidence, the model has strong potential for resilience-based risk and impact assessment. Although resilience assessments are resource demanding (Spears et al. 2015), monitoring efforts of ecosystems are mounting and data are increasingly becoming available that may allow for the development and implementation of such an approach, particularly at the intersection between science, policy and management (Garmestani and Benson 2013).
